# Supplementary material for: Polyketide synthase gene clusters in the ericoid mycorrhizal fungus Oidiodendron maius: genome-wide prediction, expression profiling, and functional characterization of knockout mutants
Source: Front Plant Sci. 2026 Sep 16;17:1924430. doi: 10.3389/fpls.2026.1924430 (PMC13623903; doi:10.3389/fpls.2026.1924430)
Supplement: Supplementary file 1 [file SupplementaryFile1.zip › Supplementary Materials/Supplementary Folder 1.zip!/Oidma1_AssemblyScaffolds_Repeatmasked/knownclusterblast/cluster36/jgi.p_Oidma1_148143_mibig_hits.html]

| MIBiG Protein | Description | MIBiG Cluster | MiBiG Product | % ID | % Coverage | BLAST Score | E-value |
| --- | --- | --- | --- | --- | --- | --- | --- |
| ACD39760.1 | cytochrome\_P450 | BGC0000077 | Hypothemycin\_biosynthetic\_gene\_cluster | 40 | 98.5858585859 | 341 | 1.3e-93 |
| ACD39751.1 | cytochrome\_P450 | BGC0000076 | Hypothemycin\_biosynthetic\_gene\_cluster | 40 | 98.5858585859 | 341 | 1.7e-93 |
| BAD83682.1 | cytochrome\_P-450 | BGC0000012 | Alternapyrone\_biosynthetic\_gene\_cluster | 37 | 98.5858585859 | 337 | 2.5e-92 |
| BAK26559.1 | cytochrome\_P450 | BGC0000977 | Cyclopiazonic\_acid\_biosynthetic\_gene\_cluster | 37 | 99.595959596 | 331 | 1.8e-90 |
| AAK33080.1 | putative\_trichothecene\_C-15\_hydroxylase | BGC0001277 | Nivalenol\_/\_deoxynivalenol\_/\_3-acetyldeoxynivalenol\_/ | 38 | 100.0 | 327 | 3.4e-89 |
| AAK53587.1 | isotrichodermin\_C-15\_hydroxylase | BGC0000931 | Trichothecene\_biosynthetic\_gene\_cluster | 38 | 100.0 | 326 | 5.8e-89 |
| BAE56591.1 |  | BGC0001123 | Aspirochlorine\_biosynthetic\_gene\_cluster | 40 | 99.3939393939 | 325 | 1.3e-88 |
| AAK33070.1 | trichothecene\_C-15\_hydroxylase | BGC0001278 | Nivalenol\_/\_deoxynivalenol\_/\_3-acetyldeoxynivalenol\_/ | 37 | 100.0 | 325 | 1.3e-88 |
| AAK53580.1 | isotrichodermin\_C-15\_hydroxylase | BGC0000930 | Trichothecene\_biosynthetic\_gene\_cluster | 38 | 100.0 | 322 | 1.1e-87 |
| OAQ83755.1 | isotrichodermin\_C-15\_hydroxylase | BGC0001358 | Leucinostatins\_biosynthetic\_gene\_cluster | 37 | 99.595959596 | 319 | 9.2e-87 |
| AAS90058.1 | AvnA | BGC0000009 | Aflatoxin\_biosynthetic\_gene\_cluster | 38 | 94.9494949495 | 316 | 4.6e-86 |
| AAS90010.1 | AvnA | BGC0000007 | Aflatoxin\_biosynthetic\_gene\_cluster | 37 | 95.5555555556 | 316 | 7.8e-86 |
| AAS90078.1 | AvnA | BGC0000010 | Aflatoxin\_biosynthetic\_gene\_cluster | 37 | 94.9494949495 | 315 | 1.3e-85 |
| AAS90101.1 | AvnA | BGC0000006 | Aflatoxin\_biosynthetic\_gene\_cluster | 37 | 94.9494949495 | 313 | 3.9e-85 |
| AAS90032.1 | AvnA | BGC0000008 | Aflatoxin\_biosynthetic\_gene\_cluster | 37 | 94.9494949495 | 312 | 6.6e-85 |
| BAE71325.1 | cytochrome\_P450\_monooxygenase | BGC0000004 | Aflatoxin\_biosynthetic\_gene\_cluster | 36 | 94.9494949495 | 305 | 1.4e-82 |
| AAC49196.1 | putative\_p450\_monooxygenase | BGC0000152 | Sterigmatocystin\_biosynthetic\_gene\_cluster | 36 | 91.7171717172 | 301 | 1.5e-81 |
| ACH72905.1 | AflG | BGC0000011 | Aflatoxin/sterigmatocystin\_biosynthetic\_gene\_cluster | 36 | 93.3333333333 | 299 | 7.5e-81 |
| XP\_002384857.1 | cytochrome\_P450,\_putative | BGC0001234\_c2 | 4,4'-piperazine-2,5-diyldimethyl-bis-phenol\_biosynthetic\_gene | 36 | 101.414141414 | 290 | 3.5e-78 |
| BAQ25465.1 | cytochrome\_P450 | BGC0001280 | Betaenone\_C\_/\_betaenone\_A\_biosynthetic\_gene\_cluster | 33 | 99.797979798 | 282 | 1.2e-75 |
| ACD39773.1 | cytochrome\_P450 | BGC0000134 | Radicicol\_biosynthetic\_gene\_cluster | 34 | 97.9797979798 | 272 | 7.6e-73 |
| EFY94501.1 | benzoate\_4-monooxygenase\_cytochrome\_P450 | BGC0000337 | Destruxins\_biosynthetic\_gene\_cluster | 33 | 98.5858585859 | 270 | 4.9e-72 |
| BAD29972.1 | P450\_monooxygenase\_1 | BGC0000676 | Aphidicolin\_biosynthetic\_gene\_cluster | 34 | 98.3838383838 | 267 | 2.4e-71 |
| Bcin12g06370.1 |  | BGC0000631 | Botrydial\_biosynthetic\_gene\_cluster | 33 | 94.9494949495 | 243 | 6.4e-64 |
| AGK82824.1 | cytochrome\_P450-2 | BGC0001323 | Monoterpenes-diterpenes\_biosynthetic\_gene\_cluster | 23 | 86.6666666667 | 105 | 1.6e-22 |
| AGK82817.1 | cytochrome\_P450-1 | BGC0001324 | Monoterpenes-diterpenes\_biosynthetic\_gene\_cluster | 23 | 92.3232323232 | 100 | 5.1e-21 |
| AGK82807.1 | cytochrome\_P450-1 | BGC0001322 | Lycosantalonol\_biosynthetic\_gene\_cluster | 23 | 104.444444444 | 98 | 2.5e-20 |
| ADD83003.1 | PtnO2 | BGC0001156 | Platencin\_biosynthetic\_gene\_cluster | 32 | 38.5858585859 | 79 | 9.4e-15 |
| chr3.CM0292.110.r2.m |  | BGC0001317 | Lupeol\_biosynthetic\_gene\_cluster. | 23 | 96.5656565657 | 77 | 3.6e-14 |
| ACO31275.1 | PtmO2 | BGC0001140 | Platensimycin\_/\_platencin\_biosynthetic\_gene\_cluster | 31 | 38.5858585859 | 77 | 6.1e-14 |
| ALA62324.1 | methylphloroacetophenone\_synthase | BGC0001309\_c2 | Usnic\_acid\_biosynthetic\_gene\_cluster | 22 | 78.7878787879 | 76 | 1.4e-13 |
| AAC01737.2 | putative\_cytochrome\_P450\_monooxygenase | BGC0000136 | Rifamycin\_biosynthetic\_gene\_cluster | 24 | 71.3131313131 | 55 | 2.5e-07 |
